# Significance of CD44 and CD24 as Cancer Stem Cell Markers: An Enduring Ambiguity

**DOI:** 10.1155/2012/708036

**Published:** 2012-05-30

**Authors:** Appalaraju Jaggupilli, Eyad Elkord

**Affiliations:** ^1^Biomedical Research Centre, School of Environment & Life Sciences, University of Salford, Salford M5 4WT, UK; ^2^Department of Medical Oncology, School of Cancer and Enabling Sciences, The University of Manchester, Manchester Academic Health Science Centre, Manchester M20 4BX, UK

## Abstract

Cancer stem cell population is a subset of cells capable of dictating invasion, metastasis, heterogeneity, and therapeutic resistance in tumours. Eradication of this rare population is a new insight in cancer treatment. However, prospective identification, characterization, and isolation of these CSCs have been a major challenge. Many studies were performed on surface markers for potential identification and isolation of CSCs. Lack of universal expression of surface markers limits their usage and no best combination of markers has yet been confirmed to identify CSCs capable of initiating and metastasizing tumours. CD44, a hyaluronic acid receptor, is one of the most commonly studied surface markers, which is expressed by almost every tumour cell. CD24, a heat stable antigen, is another surface marker expressed in many tumour types. However, their expression and prognostic value in isolating CSCs are still an enduring ambiguity. In this critical review, we assess the role of CD44 and CD24 in tumour initiation, development, and metastasis. We mainly focus on analysing the significance of CD44 and CD24 as CSC surface markers in combination or with other putative markers in different types of cancer.

## 1. Introduction

Cancer stem cells (CSCs) can be defined as a population of cells present in tumours, which can undergo self-renewal and differentiation. Similar to normal stem cells, CSCs can also give rise to all cancer cells in a tumour and hence termed cancer stem cells. Overwhelming evidence supports the vital role of this subset of cells in initiation and maintenance of a tumour in addition to their capability to dictate invasion, metastasis, heterogeneity, and therapeutic resistance in tumours. Identification and isolation of these CSCs using putative surface markers have been a priority of research in cancer. However, definition of specific CSC surface markers in all cancer types requires further investigations. It is clear that heterogeneity amongst tumours and within tumour subtypes renders it difficult to discover unique markers. Surface markers exhibit variable expression levels at different stages of tumour while their key regulatory functions remain unclear. However, with advancement of knowledge in this concept, the well-accepted cancer stem cell surface markers are CD44, CD24, CD133, CD166, EpCAM, and so forth, in different tumours including breast, lung, pancreas, prostate, colorectal, renal, and ovarian, while the prognostic value of these markers is still under investigation. Although cells in tumours expressing these markers possess stem cell characteristics, the question is whether they have true potentials to initiate and metastasize tumours. Therefore, alternate speculation is CSCs may not be termed as tumour-initiating cells. Thus, it demands urgent need of specific markers that can distinguish and target these CSCs. No best combination of markers has yet been confirmed to identify CSCs that are capable of initiating and metastasizing tumours. Several stem cell surface markers and biomarkers are being exploited in various cancers to determine a principal pattern of CSC markers. Proper screening and profiling of each marker with respect to each tumour type and tumour subtype is very vital in this process.

CD44 and CD24 have been used extensively in combination or with other putative markers to isolate CSCs from solid tumours [1, 2]. However, the lack of their universal expression limits their usage to few designated cancer types. Owing to current contradictions, we herein review recent literature and discuss the importance of CD44 and CD24, as potential surface markers in identification and isolation of CSC, in different cancers. We also discuss CSCs differential proportionalities in various cancer cell lines and mechanisms involved in interconversion of CD44 and CD24 phenotypes.

## 2. Cancer Stem Cell Concept: Primary View

Cancer stem cell (CSC) concept is an exciting area of research in cancer that generates a pronounced avenue to unravel and exploit novel strategies for treating cancer. Over a decade ago, studies on acute myeloid leukaemia had pioneered the CSC concept from CD34^+^CD38^−^ phenotype [3, 4]. With subsequent studies [5, 6], the existence of CSC in solid tumours is now acceptable with distinct phenotypic and functional abilities to generate tumours in xenograft models [7, 8]. These are also termed as tumour-initiating cells, which are not unanimously accepted [9]. CSCs exhibit efficacy in tumourigenesis, metastasis, and therapeutic resistance [10]. Eradication of this rare population is a new insight in cancer treatment [11, 12], but prospective identification, characterization, and isolation of these CSCs have been a major challenge. Heterogeneity amongst tumours and tumour cells [13], complex mechanisms and lack of specific markers to target them are the frontline hurdles in CSC theory [14–16].

Moreover, the origin of CSCs is debatable and uncertainty still continues. The major question is how these cells could maintain self-renewability with specific differentiation pathways [12]. Some postulations were made that normal stem cells and their progenitors could manifest CSCs activity as a consequence of accumulated mutations [9, 17]. But this may not be the sole source and cannot be applied to all CSCs [18]. If the signalling and molecular pathways were considered from normal stem cells, CSCs seem to have a possible connection with Wnt, Hedgehog (Hh), and Notch signalling pathways. Aberrant mutations due to several environmental factors and carcinogens such as cigarette smoke, radiations, and reactive oxygen species (ROS) [19] may cause reprogramming of epigenetic machinery and extensive changes in the DNA [20] that can deregulate the genes involved in these signalling pathways [21]. Consequently, the hyperactivation of these pathways is believed to cause tumourigenesis [22]. Moreover, the heterogeneity amongst tumour cells also indicate the possibility of different mechanisms in these signalling pathways where several arguments still exist [12]. Likewise in normal stem cells, it was reported that CSCs also contain lower levels of ROS associated with higher expression of ROS scavengers. Targeting ROS scavengers may induce the alterations in CSC signalling cascades [23]. Therefore, the correlation and cross-talk between transcriptional factors and protein molecules involved amongst these embryonic signalling pathways in tumour microenvironment may need extensive understanding [24, 25]. More detailed studies could give a clear picture of molecular pathways and reveal the origin of CSCs to help developing strategies for novel therapeutics in cancer.

## 3. An Unsettled Race amongst CSC Markers

There are some putative stem cell markers that are in major use for identification and isolation of CSCs from different solid tumours [13, 15, 26]. CD44 is considered a potential CSC marker in majority of cancers [15]. CD24 is another important marker whose prognostic value and significance remains controversy [7, 27]. CD24 has been investigated in combination with CD44 and other markers in various cancers. Following the identification of CSCs in breast cancer [28], these two markers gained a considerable interest to study their significance as CSC surface markers in other cancers. Though the currently available markers look promising, they are not specific to all cancers and within cancer subtypes [7, 29]. These markers may work efficiently in combination with different markers but their suitable counterparts are yet to be confirmed. Nonetheless, emerging evidences are continuing to explore their functional properties and mechanisms in tumour cells. The exploitations of these markers are still providing many interesting postulations yet to be concluded.

## 4. Molecular Functionalities of CD44 in Normal and Cancer Cells

CD44 is a multifunctional class I transmembrane glycoprotein [30] generally acts as a specific receptor for hyaluronic acid, promoting migration in normal cells and highly expressed in almost every cancer cell in its standard or variant form [31]. It is mainly associated with proteins that monitor the extracellular changes and critical in regulating cell adhesion, proliferation, growth, survival, motility, migration, angiogenesis, and differentiation [15, 30]. Also, CD44 presents cytokines and chemokines to their complimentary receptors on the cellular membrane [30]. CD44 interacts with osteopontin and regulates its cellular functions leading to tumour progression [32]. It even interacts with collagen, laminin, and fibronectin where their physiological function is unclear [31]. CD44 functions involve ligand binding receptor, coreceptor and organizer in cortical actin skeleton [30]. CD44 is expressed on cancer cell surface and assist haematogenous spread while interacting with P- or L-selectins [33]. It is also involved in numerous complex signalling cascades enhancing tumour initiations by interacting with neighbouring receptors like tyrosine kinase [34].

Though the above-mentioned functions are physiological activities in normal and stem cells, they are in turn exhibited in cancer cells as well. CD44 gene often undergoes alternative splicing to encode different proteins in different cancer subtypes [32], displaying its multifaceted expression. Hence, CD44 is extensively used as a surface marker for isolating CSCs from breast, prostate, pancreas, ovarian, and colorectal cancers [15, 35]. In combination with other surface markers, CD44 can also discriminate between a variety of cancer subsets [36]. As few as 100 cells, CD44^+^cells promoted tumourigenesis in breast, and colorectal cancer displaying stem cell properties such as self-renewal and differentiation.

However, there are contradictions in validating CD44 expression level and correlation with disease prognosis. For several years, the usefulness of CD44 as CSC marker has been uncertain [1]. In many cancers, CD44 plays a major role in initiation [26], metastasis [29, 37, 38], and promoting tumourigenesis [39]; while other studies opposed this relationship in other human cancers like breast [40] and prostate [41] cancers, where its high expression also showed no carcinogenesis [42]. CD44 is expressed in almost all normal and cancer cells leading to discrepancy and reflecting the ambiguity regarding functional aspects of CD44 in CSC maintenance and mechanisms involved in cross-talk with expression of stemness genes [15].

## 5. Molecular Functionalities of CD24 in Normal and Cancer Cells

CD24 is a small cell surface protein molecule anchored by glycosyl-phosphotidyl-inositol in a wide variety of cancer cells. It is heavily glycosylated and functions in cell-cell and cell-matrix interactions [2, 43, 44]. CD24 was discovered in mice as a heat-stable antigen and was used as a marker to differentiate hematopoietic cells and neuronal cells [45, 46]. Variable glycosylation on CD24 attributes to distinct functions in different cells in which some are still unclear. Due to its distinct glycosylation, it acts as a versatile ligand in various cells including cancer cells with diverse physiological functions, making its mechanisms more complex to understand [46].

CD24 is highly expressed in ovarian, breast, prostate, bladder, renal, nonsmall cell carcinomas, and other human cancers [43, 47]. It is involved in cell adhesion and metastasis [2]. This indicates that CD24 could be a significant marker in tumour prognosis and diagnosis. Functionally, it is identified as an alternate ligand for P-selectin, an adhesion receptor on platelets and endothelial cells [48], through which their interaction facilitates the passage of tumour cells in blood stream during metastasis. It increases proliferation and adhesion of tumour cells to fibronectin, collagen, and lamin [47]. The metastatic associations of CD24 increase its importance as a prognostic factor and a new CSC marker [2].

Recent investigations found that CD24 is coexisting with CD44, CD29, and CD31 in various cancers and gained new interest as a CSC marker. High expression of CD24 is involved in tumour progression [2] and metastasis [29], while the intracellular studies in pancreatic cancer [49] showed CD24 expression both on membrane surface and intracellular environment but inhibited the cell invasion and metastasis. This indicates the distinct expression and function of CD24 in different cancers. Also CD24 appears to possess less expression in progenitor cells when compared to differentiated cells. Hence, studies are required on its underlying mechanisms in both differentiated and undifferentiated cells [46].

## 6. Are CD44 and CD24 Reliable CSC Markers?

**Figure 1:**
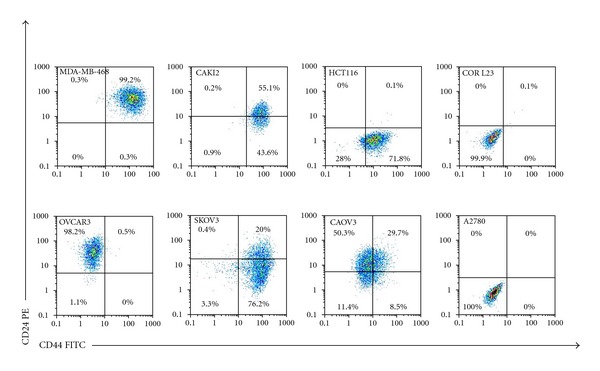
FACS analysis for double staining of CD44 and CD24 in selected human cancer cell lines. These cell lines are breast (MDA-MB-468), renal (CAKI2), colon (HCT116), lung (COR L23), and ovarian cancer (OVCAR3, SKOV3, CAOV3 and A2780). Each cancer cell line shows differential expression for both CD44 and CD24. It is highly variable even amongst cell lines of same cancer types indicating the heterogeneity between tumours.

Many studies refer to CD44 as a commonly expressed surface marker in different cancer types. The majority of cancer cell lines express high levels of CD44. Differently and in spite of its expression in many different cancer subtypes, the ambiguity on CD24 classification and distribution is still persisting. The conclusions from several studies regarding their expression, role in tumour initiation, and metastasis, and membrane distribution appear to be different [26, 50–53]. However, including CD44 and CD24, no marker can be used universally to identify CSCs in various cancers. It is certainly acceptable that these markers are not expressed in all cancers. We found that the levels of CD44 and CD24 expression show great variation (Figure 1) between cell lines even in cells of the same cancer subtype [53]. They are engaged with distinct functionalities at different time periods during tumour progression and metastasis [18]. It strongly infers heterogeneity between and amongst cancer subtypes, which is not fully elucidated [50] and raises a question of credibility regarding their value as CSC surface markers. Despite the extensive study and emerging evidence, significance of CSC markers, their specificity, correlation, and coexistence remain elusive. Also, enormous data from literature is leaving ambiguity even for the same type of cancer subtypes and CSC markers. Collectively, CSC concept seems to be left more complicated with complex implications yet to be resolved.

## 7. Proportions of CSCs and Expression Level of CD44 and CD24 Markers

Initially CSCs were considered to be a very rare subset of cells in tumours characterized with stem cell properties and tumour initiating capability [18]. According to early studies, it was 0.1%–1% in leukaemia and 2% in breast cancer [4, 5, 28], which was later supported by many scientists. Recent studies in colorectal cancer cell lines showed 0.5%–1% of CD44^+^/CD24^+^ cells gave rise to the highest proportion of crypt-forming megacolonies and differentiated to all combinations of CD44^±^/CD24^±^ [8]. More recently, Ricardo et al. [7] demonstrated the frequency of CD44^+^/CD24^−/low^ as ≥10% in basal-like breast tumours. In contrast, a recent mathematical model proposed that CSCs could be any possible proportion of the tumour and its tumourigenesis is directly proportional to the number of CSCs [54].

However, studies on cancer cell lines showed substantial variation in CD44 and CD24 expression. Study on NC160 tumour cell lines panel by Stuelten et al. [53] found that CD44 and CD24 expression is highly varied even amongst same tumour type cell lines. In support of these results, another study in ovarian cancer observed great variation in CD44 and CD24 expression in almost all patients ranging from 2.2%–88.2% and 3.2%–86.7%, respectively, [55]. However, there is a discrepancy with the results amongst other scientists. Subsequent studies by Leung et al. [26] and others claimed that their results differ from Stuelten and colleagues. Also, in our preliminary study on selected cancer cell lines, we observed a range of variation in CD44 and CD24 expression even amongst same cancer types (Figure 1). We also observed that data differs in these two studies. The persisting inconsistency amongst emerging data consequently may mislead the conclusions. One of the main reasons for this difference from study to study could be the variation in selected samples. Data from individual cell in culture may vary from primary tissue or colony-forming cells or transition cells due to distinct morphological and microenvironmental properties [18]. For instance, a recent study on cells from primary ovarian carcinoma in different patients expressed CSC surface markers at different levels [55]. Therefore, until now, there are no precise proportions of CSCs or individual markers capable of initiating and metastasizing tumours.

## 8. Correlation between CD44, CD24 Expression and Metastasis

With advancement of studies in CSCs, the correlation between marker expression, tumour initiation, invasion, and metastatic properties has been questioned [8, 56]. Furthermore, coexpression of surface markers in CSCs is sometimes debatable in several cancer types. Every marker shows independent expression level but seems to have coordination with each other in developing tumours at different stages. A recent study demonstrated no correlation between marker expression and tumourigenic potentiality in CSCs of the same cancer type obtained from different patients [55].

Abraham et al. [57] demonstrated ≤10% prevalence of CD44^+^/CD24^−^ in 78% and >10% of these cells in 22% of breast cancer samples and extended to suggest that there is no correlation between the prevalence of CD44^+^/CD24^−^ cells and clinical outcome but indicated the chance of metastasis. In contrast, Sheridan et al. [56] demonstrated that there is no correlation between the prevalence of CD44^+^/CD24^−^ cells with distant metastasis. This phenotype is associated with invasive properties but lacks the ability to proliferate. Supporting these two findings, Fillmore and Kuperwasser [58] also showed that there is no correlation between the percentage of CD44^+^/CD24^−^ cells and tumourigenicity. Later on Choi et al. demonstrated that CD133, CD24, and CD44 expression is related to invasiveness and differentiation but did not show a close relationship with the survival outcome of colorectal adenocarcinoma [59]. Stuelten et al. [53] also showed no correlation between the marker expression and their clonogenic potentiality in cell lines. But very recently, and for the first time, a study in colorectal cancer revealed coexistence of CD133^+^ CD44^+^ stem-like cells with CD133^+^CD44^high^ subset that exhibited more invasive *in vitro* and metastasis to liver *in vivo* [60]. However, direct investigations on metastatic ability of CSCs *in vivo *are very few and more validations are required. Significantly, it was found that CSCs express invasive genes *in vivo *but are incapable of metastasis [61].

## 9. Strategies Implemented on CD44 and CD24 to Evaluate Their Significance as CSC Surface Markers

It is highly convincing that tumour recurrence can be prevented if CSCs are specifically targeted. Development of drugs to target and eradicate CSCs represents a major challenge due to the fact that they are resistant to conventional therapies. The slow replication rate of cancer stem cells compared to adult cancer cells and overexpression of ABC transporter proteins that are capable of eliminating the molecules used in cancer therapies could be possible reasons for inherent resistance in CSCs [59, 62]. Regulation of CD44 or CD24 expression in various cancers seems to be effective in controlling tumour initiation and inhibition of CSC population. Jin et al. [63] demonstrated reduction of leukemic stem cells in acute myeloid leukaemia by targeting CD44 function. Therefore, CD44 could be a potential therapeutic target in solid tumours that are expressing this molecule. However, the influence of alteration in CD44 function on normal stem cells has to be considered during experiments on a cancer patient [29].

Genetic manipulations of certain surface marker expression in CSCs and examining the effect on cancer progression have been under investigation. MicroRNA (miRNA) has been used to downregulate specific gene functions in CSCs and repress their expression in order to understand their key functional activities. They are also being used to inhibit proliferation and induce apoptosis, senescence when transfected into cancer cells [64]. By using these approaches, inducing the differentiation of CSCs may transform them to mature cancer cells which can be more vulnerable to conventional therapies [65]. This miRNA could be advantageous in treating malignant tumours by repressing specific genes of signalling pathways involved in CSCs [11]. Previous studies [66, 67] demonstrated these strategies. Yu et al. [68] work with let-7 miRNA in breast-cancer-induced CD44^+^/CD24^−/low^ cell population to differentiate and consequently inhibited formation of tumours. Using mullerian-inhibiting hormone, Wei et al. [69] demonstrated inhibition of stem/progenitor cell population in tumours. More recently, Liu et al. [64] showed that miR-34a inhibits prostate cancer stem cells and metastasis by directly repressing CD44 expression. However, widespread explorations of miR-34a functionalities are necessary for complete understanding and could be considered for further therapeutic implications [70]. Recent studies on CD24 depletion using siRNA and NDRG2 regulation in hepatocellular carcinoma [47, 71] and shRNA methods in bladder cancer [72] demonstrated the reduction of tumourigenesis and CD24 could be used as a prognostic marker. However, absence of CD24 expression in breast and prostate cancer restricts its use as a potential target for controlling invasion and metastasis [73]. This suggests that CD24 expression seems to be strictly tissue specific and more elucidations necessary to develop more obvious therapeutic strategies. However, the miRNA application has to be viewed as a strategy to analyse the expression and regulatory function of a particular gene in different cell types. At this context, it may be helpful in understanding the role of a surface marker in driving tumour initiation and metastasis of different tumour types. Knock-down of CD44 or CD24 may enhance CSC differentiation to mature cells and reduce tumour progression [74]. Targeting key regulatory genes in CSCs may control their critical signalling pathways, but it has to be specific to targeting genes expressed in only CSCs but not normal stem cells [29].

## 10. CD44^+^/CD24^−/low^ as Breast CSC Marker

After pioneering studies of Al-Hajj et al. [28] in human breast cancer, CD44^+^/CD24^−/low^ cells were recognized as prospective cancer stem cells for basal/mesenchymal cell lines MCF 7 and MDA-MB-231. This was subsequently supported by Sheridan et al. [56] but suggesting this phenotype alone may not be appropriate to envisage metastasis though these cells are rich in invasive genes. Later on, large body of evidence demonstrated that this phenotype is not expressed in all breast cancers emphasizing the need for identification of other breast CSC markers [7, 50, 51].

MDA-MB-468 cell line exhibits high expression of CD44^+^/CD24^+^ cells with basal/epithelial phenotype [7]. We found similar results in our flow cytometric studies on this cell line (Figure 1). It is highly accepted that CD44^+^/CD24^−^ exhibit undifferentiated basal/mesenchymal cell properties and CD44^+^/CD24^+^ exhibit highly differentiated basal/epithelial cell properties [7]. But how epithelial cell lines like MDA-MB-468 with high CD24 expression could induce tumours remains unclear. Though MDA-MB-468 is rich in CD44^+^/CD24^+^ cells that are not invasive, it is believed that few CD44^+^/CD24^−^ cells in this cell line are progressed to CD44^+^/CD24^+^phenotype after metastasis which seem to have an interconversion of CD24^+^ to CD24^−^ [56]. Recent studies are evident for migration, colony formation, and invasion in MDA-MB-468 by CD44^+^ cells that give support to the CD24 interconversion and regulatory concept [75]. Shipitsin and Polyak [76] hypothesized relationship between CD44^+^ and CD24^+^ genotypes and their origin. In another study, Honeth et al. [50] found predominant expression of CD24 in cytoplasm along with CD44 high expression on surface membrane, but disturbances in protein distribution or its degradation may regulate enrichment of CD24 on membrane. It seems that few alterations may take place in regulating the level of CD24 expression on membrane surface during tumour initiation and metastasis. Subsequently, Meyer et al. [77] supported the possibility of interconversion between the phenotypes and suggested that epithelial like CD44^+^/CD24^+^can readily give rise to CD44^+^/CD24^−^ cells during tumour initiation. Also, these studies supported the possibility for CD24^+^ origin from CD44^+^ cells indicating they are genetically identical [29]. Based on the same concept, recently, Shi et al. [78] also demonstrated in human epithelial ovarian cancer, that CD44^+^/CD24^−^ cells showed CSC-like properties and differentiated to CD44^+^/CD24^+^. A recent study by Ricardo et al. [7] has indicated more additional questions that to be elucidated. Until recently ALDH1 was considered as one of the potential markers for identifying breast cancer stem cells along with CD44^+^/CD24^−/low^. It seems CD44^+^/CD24^−/low^ phenotype and high ALDH1 expression overlap at significantly low proportion of 1% [79]. Ricardo et al. concluded that little overlap between these two markers limits their prognostic value even amongst basal-like subtypes in breast cancer indicating the distinct differentiation amongst same cancer subtypes.

Regardless of the positivity or negativity of CD44 and CD24, the cells expressing these markers in breast cancer were highly acknowledged with invasive properties but lacking metastatic genes [56]. Although many studies proved the vital role of CD44 in metastasis [34, 80], contrastingly, Lopez et al. [40] demonstrated that CD44 can inhibit metastasis in the breast cancer. On the other hand, activity of CD24 was also having the similar attention with contradictory explanations. Baumann et al. [81] showed CD24 promotes invasion, while Schabath et al. [52] demonstrated expression of CD24 inhibiting the invasion and metastasis. Conversely, the prognostic value of these markers in breast cancer is still a controversy, which needs more research to disclose these complications [7, 27]. Therefore, these markers cannot be referred as supreme markers for identifying breast cancer stem cells [9].

## 11. Ambiguity over CD44 and CD24 Expression in Ovarian Cancer

The variations in CD44 and CD24 proportions from cell line to cell line show the phenotypic heterogeneity in different cancer types (Figure 1). Our data represent similar results with past evidences and indeed contrasting with some other studies too. Nevertheless, there is a great demand for more intensive studies in addition to current investigations of CSCs in various cancer types. This notion of identifying potential CSCs has also directed its extensions to ovarian, prostate, pancreas, colorectal, renal, and lung cancers. CD44 and CD24 have been studied extensively to determine their significance as CSC markers. Apart from CD44 expression, CD24^+^ cells are also reported to act as cancer stem cells in ovarian and colorectal cancers [8]. Therefore, the significance of CD24 expression cannot be overruled.

Ovarian cancer cell lines show high expression of CD24 as they have epithelial phenotype [82]. With critical review of literature, it appears that the importance of CD44 and CD24 expression in ovarian cancer is more debatable. It is realized that there are no potential markers to detect ovarian cancer at an early stage and they are not studied completely [35, 83]. From initial studies [84] in human ovarian cancer, both side population (SP) and non-SP in one cell line (MOVCAR8) showed high expression of CD44 whereas other human ovarian cancer cell lines do not express CD44. In the same study, the authors indicated that CD24 showed variable expression in these cell lines. Following these studies, Zhang et al. [85] demonstrated that CD44^+^CD117^+^ cells have the potential to initiate epithelial ovarian cancer, which was later supported by other studies as well [55, 86, 87]. Additionally, Slomiany et al. [1] reported that CD44^+^CD117^+^ phenotype has a prognostic value in combination with CD133. But in contrast, a recent study [88] reported that CD117 is not enriched in most of the ovarian cancer cell lines. Although CD44 appears to be a promising marker in several ovarian cancer cell lines, it cannot be used solely, and other potential markers should be combined in order to characterise and isolate CSCs.

According to published data on different ovarian cancer cell lines, there appear to be substantial variations in proportions of CD44 and CD24. For instance, CD44 expression is high in SKOV3 but no expression found in OVCAR3 [36, 89, 90], which is similar to our results (Figure 1). Previous studies also demonstrated the CD44 expression in SKOV3 and CAOV3 [91]. In contrast, there are also recent studies arguing high expression of CD44 in OVCAR3 [92, 93]. It is also the same paradigm with A2780 where Rainaldi et al. [94] and Santini et al. [95] stated the absence of CD44 in A2780, while another recent study [96] demonstrated high expression of CD44 in A2780. However, in our study, when examined A2780 cell line, neither of the markers showed positive expression. Apart from CD44, many studies refer CD24 with vital role in ovarian cancer metastasis, and it can be used as a potential CSC marker [69, 89, 97, 98]. Recently, Gao et al. [97] demonstrated that 5000 CD24^+^ cells could form tumours in animal models with high expression of stemness genes, while the same number of CD24^−^ cells showed nontumourigenic efficiency. We found that 20% of CD44^+^ SKOV3 cells expressed CD24, while CAOV3 showed lower expression of CD44 (40%) and higher expression of CD24 (80%) see Figure 1. Most OVCAR3 cells expressed CD24 with lack of CD44 expression (Figure 1). This indicated that OVCAR3 is more of epithelial phenotype compared to other cell lines. Nevertheless, unless these cells from each cell line were isolated and studied through xenograft experiments, their tumourigenic ability cannot be concluded.

In their study, Stuelten et al. [53] demonstrated CD44^−^ cells generated both CD44^+^ and CD44^−^ phenotypes in OVCAR5 cell line reflecting its stem cell properties. Although such consequence is contrasting with CD44^+^ cells in breast cancer [28], eventually it has shown only CD44^+^ cells possess greater clonogenic ability than CD44^−^ cells. This may strongly support the interconversion of CD44 phenotype during tumourigenesis, indicating the differential expression of markers in tumours. However, CD44 is expressed in almost all cancers, likewise, recent reports show CD24 is also enriched in ovarian CSCs. Wei et al. [69] demonstrated that CD44^+^CD24^+^EpCAM^+^ phenotypes in human ovarian cancer cell lines are enriched for stem/progenitor cells clonogenic ability. Clearly, the notable marker combination is yet to be confirmed.

## 12. CD44/CD24 in Prostate and Pancreatic Cancers

The CSC research in prostate and pancreatic cancers remains fresh. Only recently, some potential markers were elucidated and found few populations comprising tumour-initiating ability. In pancreatic cancer, CD44^+^/CD24^+^ was demonstrated as potential phenotype to isolate CSCs [99]. Later, CD44^+^/CD24^+^/ESA^+^ cells were referred to CSCs with 0.5%–1% of all pancreatic cancer cells [100]. However, Qiang et al. [101] demonstrated CD44^+^/CD133^−^ phenotype as a useful marker combination in predicting clinical outcome. On the other hand, another study reported that CD44^+^/CD133^+^ cells were enriched with tumour-initiating characteristics [102]. In pancreatic cancer initially, Collins et al. [103] found CD44^+^ *α*2*β* ^+^D133^+^ population was able to differentiate to mixed phenotype in prostate cancer. Later on, Patrawala et al. [104], using several prostate cell lines and xenograft tumours, reported high expression of CD44^+^ cells, and these cells enhanced proliferation *in vitro* and tumour initiation and metastasis *in vivo* [105]. Subsequently, Hurt et al. [106] isolated CD44^+^/CD24^−^ cells from prostate cancer and identified tumour-initiating ability in this phenotype showing clonogenic and differentiation capability. It was rather supported by other studies indicating CD44 role in tumour invasion but with no clear information about metastasis [53, 64, 107]. Thus, when compared to other cancers, the knowledge about prostate CSCs seems to be inadequate and requires further investigations to find prominent combination of surface markers [108]. These studies represent CD44 as a potential marker, but it is too ambiguous with its counterpart combination in both pancreatic and prostate cancers. As CD24 and CD133 are enriched within epithelial and differentiated cells [109], more elucidations may require to define potential marker combination.

## 13. CD44/CD24 in Colorectal, Lung, and Renal Cancers

The ambiguity exists in these cancer types as well. In colorectal cancer, Shmelkov et al. [110] reported the interconversion of CD133^+^to CD133^−^ phenotype after metastasis seems to be similar as in breast cancer. Also, they demonstrated that these two populations could initiate tumours. RNA interference (RNAi) studies of Du et al. [15] indicate that CD44 as a potential marker for CSCs in colorectal cancer and cells with high expression of CD44 along with CD133 in HCT116 showed tumour-initiating capability [60, 111]. These conclusions infer that CD44 in coexistence with CD133 may have high tumourigenic activity rather than with CD24; whereas later studies reported that CD133 was not expressed in all solid tumours and may not be considered as a counterpart of CD44. Instead, EpCAM^high^/CD44^+^/CD166^+^ was suggested as robust CSC phenotype for isolation of CSCs [13]. However, recent studies on colorectal cancer cell lines [8] demonstrated that CD44^+^/CD24^+^ cells showed greater clonogenic ability *in vitro *and tumour initiation *in vivo*. This phenotype is differentiated to all four combinations of CD44/CD24 cells. But one amongst cell lines is HCT116, which is known to be a highly aggressive cell line with little or no capacity to differentiate and known to possess high proportion of stem cell markers [13]. HCT116 expresses CD44^+^/CD24^−^ phenotype cells with less clonogenic ability [8]. This is similar to studies of Ahmed et al. [112] showing very low levels of CD24 in HCT116. Our work on HCT166 (Figure 1) also showed high expression of CD44 with no expression of CD24. However, the source of CSC in colorectal cancer is controversial and has to be elucidated completely [14].

We analyzed COR L23, a nonsmall cell lung cancer cell line that was completely negative for both CD44 and CD24 markers (Figure 1). No strong evidences showed CD44 and CD24 analysis on COR L23. CD44 is believed to be a potential marker in lung cancer but not expressed in all lung cancer types. Therefore, it may not be considered as a bona fide CSC marker. Nonetheless, the CSCs in lung cancer are still remaining to be explored which has not been studied intensively as other cancers [26]. We also considered CAKI2, which is a primary clear cell renal carcinoma cell line to compare the CD44 and CD24 expression. We found that almost all CAKI2 cells express CD44 and 55% of them express CD24. Though studies indicate high expression of CD24 and CD44 in renal cancer [113–115], more investigations with different experimental approaches are required to examine their importance as CSCs.

## 14. Conclusion

The ambiguity of CSC markers is persisting despite of large body of literature and information. Additional in-depth knowledge to the present available information is necessary to explore mechanisms involved in tumour development and maintenance. Further investigations are necessary to determine specific marker profiles to each tumour type and patterns in each cell line since it appears to have high distinction between cell lines amongst the same tumour types and different tumour types. CD44 appears to have a significant regulatory role in almost all cancer types and more elucidations on this marker may contribute to evaluate its prognostic importance. The regulatory interaction of CD44 in signalling pathways in tumour cells remains unresolved. CD24 being an epithelial marker, it may not be expressed in all cancers but seems to have a significant role in those expressing tumours. Close understanding of CSC origin and function is necessary. As all tumours have differential phenotype, it is obvious to express various specific markers in regard to the tissue origin and functional properties. Studies also indicate high variation amongst their proportions. Hence, profiling the ultimate combination of markers based on their physiological and functional properties in each tumour could help in designating a specific marker combination to each tumour subset. More intensive studies may help reducing the ambiguity amongst available information and finding potential prognostic CSC markers.
